# The longitudinal exercise trend among older Swedes aged 53–84 years – a 16-year follow-up study

**DOI:** 10.1186/1471-2458-14-1327

**Published:** 2014-12-29

**Authors:** Patrik Midlöv, Matti Leijon, Jan Sundquist, Kristina Sundquist, Sven-Erik Johansson

**Affiliations:** Center for Primary Health Care Research, Department of Clinical Sciences, Lund University, Malmö, Sweden; Stanford Prevention Research Center, Stanford University, Palo Alto, California USA

**Keywords:** Exercise, Older adults, Longitudinal studies, Cohort effect, Mixed models

## Abstract

**Background:**

Many older adults are physically inactive and inactivity increases with age. This knowledge comes from cross-sectional studies. Cross-sectional studies may miss important trajectories within the older adults as a result of retirements, and poor health impact of promotional efforts. The aim of this study was to analyse, longitudinally, the annual effects of age group and birth cohort on self-reported regular exercise in the Swedish population aged 53–84 years during a 16-year period, for each sex separately.

**Methods:**

A random sample of non-institutionalized persons was interviewed three times from 1988 to 2004 by professional interviewers. In addition to three time-related variables – year of interview, age at the time of the interview, and year of birth – we included the following explanatory variables in the analyses: educational level, body mass index, smoking, and self-reported health status. The data were analysed by a mixed model with a random intercept.

**Results:**

The total prevalence of self-reported regular exercise increased between 1988/89 and 2004/05 among both men and women, from 27.1 to 43.1% and from 21.1 to 41.1%, respectively. There was a mean annual change in all age-groups in exercise of between 0.76 and 1.24% among men and between 0.86 and 1.38% among women. Low prevalence of self-reported regular exercise was associated with low educational level, obesity, smoking, and poor self-reported health, although those with poor self-reported health the greatest increase of physical activity.

**Conclusions:**

There was a steady, albeit inadequate, increase in self-reported regular exercise in older adults between 1988 and 2004. Physical activity promotion in older adults should be of high priority for both primary and secondary prevention of diseases, especially among groups with known risk factors for low levels of exercise.

## Background

In most parts of the world there have been rapid declines in death rates during the last 100 years leading to increases in the proportion of each birth cohort surviving beyond 65 years [1]. Inventions and new technology have contributed to these improvements in life expectancy but have also, unfortunately, promoted a lifestyle characterized by little physical activity. This means that a high proportion of elderly people are expected to be less physically active.

The health benefits of exercise and risks of inactivity have gained increased evidence in the 21st century. Advancing age is associated with increased risk for chronic diseases, but physical activity significantly reduces this risk [2]. Physical activity improves quality of life in adults with chronic conditions [3], has positive effects on coronary heart disease risk [4] and reduces the risk of falls [5]. It has been estimated that physical inactivity causes 6-10% of the major non-communicable diseases coronary heart disease, type 2 diabetes, and breast and colon cancers worldwide [6]. The benefits of physical activity are well-known [7].

Despite these facts, many older adults are physically inactive and this inactivity increases with age [2, 8–11]. These studies are cross-sectional and may miss important trajectories within the older adults as a result of retirements, and poor health impact of promotional efforts. It may be different when the same people are followed over time. Thus, there is a need for longitudinal studies on the exercise of older adults. The effect of age group and birth cohort on exercise in older adults has been shown [12] but to our knowledge only based on a series of cross-sectional studies and not in any longitudinal studies.

Several different confounders are important when studying level of exercise. Sex, educational level [13, 14], BMI [13], smoking status [15, 16], drinking problems [17], and self-reported health status [18] are all associated with level of exercise.

The aim of this study was to analyse longitudinally, based on three assessments on the same individuals at intervals of eight years, the annual effects of age group and birth cohort on exercise in the Swedish population during a 16-year period, for each sex separately. Another aim was to analyse whether any observed effects remain after adjustment for the possible confounders educational level, self-reported health, smoking, and body mass index (BMI).

## Methods

### The Swedish annual level of living survey

The Swedish Annual Level of Living Survey (SALLS) is a simple cross-sectional random sample of adult, non-institutionalized persons aged 16–84 years, taken from the Swedish Total Population Register. It is representative of the adult population of Sweden and was used as the source of data in this study. Professional interviewers from Statistics Sweden conducted face-to-face interviews, usually at the respondents’ homes [19]. The data are not publicly available and the use and analysis of the data need permission from Statistics Sweden, a government agency that produces statistics [19].

Our sample consists of 995 men and 1184 women aged 53–84 who were originally drawn randomly from the Swedish Total Population Register and were followed from 1988/89 to 2004/05.

In this study, we used data from the 1988/89, 1996/97, and 2004/05 surveys for everyone who took part at in at least one survey. New individuals aged 53–60 years were included at each of the two last occasions. All analyses shown in all tables were based on the same sample sizes shown in Tables 1 and 2 according to sex and assessment period. This study covered ages of 53 to 84 years. Participants that were missing at one occasion were only excluded from that occasion (non-response). The non-response rate varied between 20 and 25% between surveys.

**Table 1:** **The distribution (%) of the different variables by sex and year (longitudinal samples of the Swedish population from 1988/89, 1996/97, and 2004/05)**

Variable	Men			Women		
	1988/89	1996/97	2004/05	1988/89	1996/97	2004/05
**N**	995	988	930	1,184	1,127	1,006
**Age (years)**						
53-60	26.4	36.5	38.8	25.4	28.7	37.8
61-68	29.3	24.0	30.9	28.3	26.2	25.7
69-76	27.7	23.5	16.9	29.1	25.2	21.5
77-84	16.6	16.0	13.4	17.2	19.9	15.0
**Educational level**						
Low-middle	75.9	70.6	60.3	86.7	81.3	67.9
High	24.1	29.4	39.7	13.3	18.7	32.1
**BMI**						
Normal	50.1	45.6	39.3	57.0	51.6	51.0
Overweight	43.1	45.8	48.7	33.6	37.2	35.1
Obesity	6.9	8.7	12.0	9.4	11.2	13.9
**Smoking**						
Never	34.0	36.7	35.9	69.4	61.2	51.1
Former	44.3	46.6	50.3	14.4	21.9	31.5
Daily	21.7	16.7	13.7	16.2	16.9	17.5
**Self-reported health status**						
Good	63.5	66.0	68.4	55.8	55.8	60.0
Poor	36.5	34.0	31.6	44.2	44.2	40.0

**Table 2:** **Unadjusted prevalence of exercise for the different variables by sex and year of interview (longitudinal samples of the Swedish population from 1988/89, 1996/97, and 2004/05) in individuals aged 53-84 years**

Variable	Men			Women		
	1988/89	1996/97	2004/05	1988/89	1996/97	2004/05
**N**	995	988	930	1,184	1,127	1,006
**Total**	27.1	30.2	43.1	21.1	21.7	41.1
**Age (years)**						
53-60	25.0^d)^	35.3^e)^	39.1^f)^	24.5^d)^	27.1^e)^	47.0^f)^
61-68	33.8^c)^	31.9^d)^	46.0^e)^	28.2^c)^	26.7^d)^	45.9^e)^
69-76	27.6^b)^	26.4^c)^	49.7^d)^	18.2^b)^	20.8^c)^	35.6^d)^
77-84	17.8^a)^	22.0^b)^	40.0^c)^	9.0^a)^	8.1^b)^	26.0^c)^
**Educational level**						
Low-middle	25.9	26.5	40.4	19.7	20.7	36.9
High	31.0	39.2	47.3	30.4	25.9	50.5
**BMI**						
Normal	28.9	36.7	48.2	22.4	25.4	46.0
Overweight	26.9	27.4	42.4	19.8	19.2	41.0
Obesity	15.9	11.6	29.5	18.0	12.6	23.7
**Smoking**						
Never	27.5	34.4	45.4	21.8	21.3	42.6
Former	30.9	28.5	44.4	25.1	25.1	45.1
Daily	18.9	26.1	32.3	14.5	18.4	29.7
**Self-reported** **health status**						
Good	34.1	36.5	49.6	28.5	28.7	50.2
Poor	14.8	18.2	29.2	11.7	12.8	27.6

**Cohort:** ^a)^1904-11; ^b)^1912-19; ^c)^1920-27; ^d)^1928-35; ^e)^1936-43; ^f)^1944-51.

### Outcome variable

The outcome variable, exercise, comprised two levels: (0) no exercise, a little exercise now and then, or regular exercise once a week; and (1) regular exercise more than once a week. The individual’s response is based on the following question: How much do you exercise in your leisure time? The alternative answers in the 1988/89 and 1996/97 surveys were as follows: (1) I get practically no exercise at all; (2) I exercise occasionally (e.g., 1-hour walks, skiing a couple of times every year, swimming, picking mushrooms); (3) I exercise regularly about once a week (e.g., fast walks, skiing, swimming, jogging, cycling); (4) I exercise regularly about twice a week (e.g., fast walks, skiing, swimming, jogging, cycling); and (5). I exercise regularly and vigorously at least twice a week (e.g., skiing, swimming, running, cycling for quite a while, ball games). In the 2004/05 survey, the examples were excluded.

#### Explanatory variables

We included three time-related categories: year of interview, age at the time of the interview, and year of birth. We also chose to include the following explanatory: sex, educational level, BMI, smoking status, and self-reported health status.

*Year of interview* comprised three categories: 1988/89, 1996/97, and 2004/05, and including all individuals included in at least one survey.

*Age at the time of interview* was categorized into the following groups (with consideration of the 8-year intervals between measurements) to measure time trends: 53–60, 61–68, 69–76, and 77–84 years. In Tables 3 and 4, age is a continuous variable centred at 67 years (agec).

**Table 3:** **Odds ratios (ORs) with 95% confidence intervals (95% CIs) for exercise in men aged 53-84 years, obtained by applying mixed models with random intercepts that include the interaction between age and cohort, age-squared, and cohort-squared**

		Model I		Model II	
Variable	Category	OR	95% CI	OR	95% CI
*Fixed effects*					
*Rate of change*					
**Agec (Int)**	Centred at 67 years	1.013	0.990-1.036	1.016	0.993-1.040
Agec*cohortc		1.0050	1.0030-1.0074	1.0051	1.003-1.008
*Initial status*					
**Cohortc**	Centred at 1924	1.035	1.010-1.060	1.032	1.007-1.058
**Cohortc-squared**		1.0018	1.0001-1.0034	1.0019	1.0002-1.0036
**Educational level**	Low-middle			1	
	High			1.34	1.08-1.66
**BMI (kg/m** ^**2**^ **)**	Centred at 24			0.91	0.88-0.94
**Smoking**	Never			1	
	Former			1.07	0.86-1.34
	Daily			0.59	0.44-0.80
**Self-reported health status**	Good			2.68	2.14-3.36
	Poor			1	
*Random effects (unstructured)*					
Var (constant)		1.13	0.71-1.80	0.89	0.52-1.53

Model II is also adjusted for educational level, BMI, and self-reported health.

**Table 4:** **Odds ratios (ORs) with 95% confidence intervals (95% CI) for exercise in women aged 53-84 years, obtained by applying mixed models with random intercepts that include the interaction between age and cohort, age-squared, and cohort-squared**

		Model I		Model II	
Variable	Category	OR	95% CI	OR	95% CI
*Fixed effects*					
*Rate of change*					
**Agec (Int)**	Centred at 67 years	0.93	0.90-0.97	0.98	0.96-1.012
Agec*cohortc		1.017	1.011-1.024	1.009	1.006-1.011
**Agec-squared**		1.005	1.001-1.010	1.005	1.001-1.010
*Initial status*					
**Cohortc**	Centred at 1924	0.98	0.95-1.02	1.02	1.00-1.05
**Cohortc-squared**		1.008	1.005-1.012	1.005	1.003-1.006
**Educational level**	Low-middle			1	
	High			1.23	0.96-1.58
**BMI (kg/m** ^**2**^ **)**	Centred at 23			0.93	0.91-0.96
**Smoking**	Never			1	
	Former			1.01	0.78-1.30
	Daily			0.48	0.35-0.65
**Self-reported health status**	Good			2.57	2.08-3.19
	Poor			1	
*Random effects (unstructured)*					
Var (constant)		1.51	1.01-2.23	1.05	0.66-1.69

Model II is also adjusted for educational level, BMI, and self-reported health.

*Year of birth* was used to measure cohort effects and comprised the following groups: 1904–11, 1912–19, 1920–27, 1928–35, 1936–43, and 1944–51. In Tables 3 and 4, year of birth is a continuous variable centred at 1924 (cohort).

*Sex:* Separate analyses were undertaken for men and women.

*Educational level* was dichotomized as follows: (1) low-middle (compulsory school or less (≤9 years) or practical high school (i.e., vocational school, 10–11 years)); and (0) high (theoretical high school and/or college (≥12 years)).

*BMI*, calculated as weight (kg) / height^2^ (m^2^), was both included as a continuous variable centred at 24 in men and 23 in women, and categorized as normal (<25), overweight (25–30), and obesity (>30) in the descriptive tables. Weight and height were self-reported. People with a missing value for either weight or height were excluded from the dataset, in total 147 missing in BMI.

*Smoking habits* were divided into three groups: (1) Never smokers; (2) Former smokers (regardless of when they quit); and (3) Daily smokers.

*Self-reported health status* was dichotomized into: (1) Good (those whose self-reported health was good in 1988/89 or good or very good in 1996/97 and 2004/05); and (0) Poor (all others).

### Statistical analysis

In the analysis, descriptive statistics were used to present the distributions of the explanatory variables (Table 1) as well as the prevalence of exercise according to the explanatory variables (Table 2) by sex and period of time.

Two mixed logistic models with random intercepts were applied to test the change over time in exercise according to age group and cohort. Including random slopes did not improve the model. Age was centered at 67 years (agec) and cohort was centered at 1924 (cohortc). Model I included agec, cohortc, and the interaction agec-by-cohortc, agec_squared (only women), and cohortc_squared. Model II was also adjusted for all explanatory variables except smoking (which was excluded due to convergence problems) (Tables 3 and 4, for men and women, respectively). The effect of time period does not need to be estimated in a longitudinal panel study, as age and time are the same variable. Instead, the focus can be on the age-by-cohort interaction. The results are presented separately by each sex as odds ratios (ORs) with 95% confidence intervals (95% CIs) and as rate of change in exercise in the different age groups (Tables 3 and 4). Least square probabilities (%) of exercise and cohort effects (change per year), based on the model in Tables 3 and 4 adjusted for BMI, education, and self-reported health status, are shown in Tables 5 (men) and 6 (women). Adjusted proportions (%) of exercise and birth cohort trends (change per birth year), as well as age trends (change per year), based on the adjusted model in Tables 3 and 4 (adjusted for educational level, BMI, and self-reported health status) are shown in Table 5 (men) and 6 (women).

**Table 5:** **Predicted prevalence (%) of exercise and annual change in exercise (Δ% per year by age and cohort, test of trend) in males aged 53-84 years, presented according to age, birth cohort, and assessment period (longitudinal samples of the Swedish population from 1988/89, 1996/97, and 2004/05) by the model in Table** 3

Variable	Age (years)					
Cohort	53-60	61-68	69-76	77-84	Δ% cohort	p-value
**1904-11**	-	-	*-*	17.4	-	
**1912-19**	-	-	25.7	**23.4**	-0.13	Ns
**1920-27**	-	31.0	***32.3***	*32.7*	0.18	Ns
**1928-35**	28.2	**34.1**	*45.6*	-	1.15	***
**1936-43**	**31.9**	*45.5*	-	-	1.51	***
**1944-51**	*39.6*	-	-	-	-	
**Δ% age**	0.76	0.97	1.24	1.00		
**p-value**	***	***	***	***		

Plain text, 1988/89; bold, **1996/97**; italics, *2004/05.*

Test for trend: ***p < 0.001; Ns, non-significant.

**Table 6:** **Predicted prevalence (%) of exercise and annual change in exercise (Δ% per year by age and cohort, test of trend) in women aged 53-84 years, presented according to age, birth cohort, and assessment period (longitudinal samples of the Swedish population from 1988/89, 1996/97, and 2004/05) by the model in Table** 4

Variable	Age (years)					
Cohort	53-60	61-68	69-76	77-84	Δ% cohort	p-value
**1904-11**	-	-	-	8.9	-	
**1912-19**	-	-	18.0	**10.4**	-0.92	***
**1920-27**	-	25.9	**20.2**	*23.0*	-0.23	**
**1928-35**	26.2	**25.7**	*34.9*	-	0.58	***
**1936-43**	**27.3**	*45.1*	-	-	1.97	***
**1944-51**	*45.9*	-	-	-	-	
**Δ% age**	1.38	1.21	1.04	0.86		
**p-value**	***	***	***	***		

Plain text, 1988/89; bold, **1996/97**; italics, *2004/05.*

Test for trend: **p < 0.01; ***p < 0.001.

Birth cohort trends (change per birth year) and age trends (change per year) were also calculated. The trends for each age group and cohort were estimated by applying a linear regression model with time as the independent variable and with the estimated proportions in Tables 3 and 4 as the dependent variable.

STATA version 12 was used for the statistical analyses [20].

### Ethics

This study was approved by the ethics committee in Stockholm (approval no. 12/2000).

## Results

Table 1 shows the distributions of the explanatory variables age, educational level, BMI, and self-reported health status, by sex and year of interview. The dataset includes new individuals aged 53–60 years who were included in the 1996/97 and 2004/05 surveys. The overall pattern shows increases in educational attainment and BMI, and improvements in self-reported health, over time.

In Table 2 the unadjusted prevalence of exercise for the different variables, by sex and year of interview, are shown. Overall, exercise tended to increase over the 16-year study period in all age groups and in both men and women. The increase was much stronger at the beginning of the new century. The prevalence of exercise increased over time among the female and male cohorts from 1988 to 2004. Persons with a higher level of education reported higher rates of exercise than those with a low level of education in all the surveys. The most dramatic relative increase in exercise was found among those reporting a poor health status. In this group, the increase over time was almost two-fold.

Tables 3 and 4 show the ORs for exercise in two mixed models with random intercepts, Model I included age, age-squared (only women), cohort, cohort-squared, and the interaction between age and cohort, while Model II was also adjusted for educational level, BMI, and self-reported health status. The adjusted model (Model II) showed similar ORs for age, cohort (significant for men), the age-by-cohort interaction (significant for men and women), cohort-squared (significant for men and women), and age-squared (only significant for women) as the initial model (Model I).

Men with a high educational level had higher odds for exercise (OR = 1.34, 95% CI = 1.08-1.66) than those with a low-middle educational level. For women there was no significant difference in odds of exercise (OR = 1.23, 95% CI = 0.96-1.58) according to educational level. The odds of exercise among those reporting a good health status were considerably higher than among those reporting a poor health status (OR = 2.68, 95% CI = 2.14-3.36 for males; OR = 2.57, 95% CI = 2.08-3.19 for females). BMI was also closely related to exercise: for every one-unit increase in BMI, the odds of physical activity decreased by 9% (OR = 0.91, 95% CI = 0.88-0.94) among men and 7% (OR = 0.93, 95% CI = 0.91-0.96) among women.

The predicted prevalence of exercise by model II in Tables 3 and 4 as well as its annual change are presented in Tables 5 (men) and 6 (women). By applying a linear regression model, we estimated the annual change by age group and cohort. In men, there was a significant mean annual change in exercise during the study period of between 1.15 and 1.51% among the two youngest cohorts. Among women, exercise increased in the cohorts from 1928 to 1943, with an annual change ranging between 0.58 and 1.97%. The age effect showed significant annual increases in all age groups of between 0.76 and 1.24% among men and between 0.86 and 1.38% among women.

## Discussion

In this longitudinal study, with three measurements on the same individuals, we found an increase of self-reported regular exercise from 1988/89 to 2004/05 totally, and in all age-groups. Among women also all cohorts increased self-reported regular exercise, while the oldest cohorts among men did not. There were, however, differences between subgroups: poor self-reported health, smoking, obesity, and low educational level are factors that were associated with low levels of self-reported regular exercise in both men and women.

These findings are in accordance with previous cross-sectional studies [15, 18]. In this longitudinal study it is clear that differences in exercise prevalence between subgroups were maintained over time. It is however noticeable that the greatest increase of physical activity was seen in those reporting poor health status. It seems that the gap in exercise between those with good and poor health status has decreased. Since physical activity is important to prevent illness this might indicate that the inequities in health will also decrease over time.

Another interesting finding was that older women increased their exercise whereas older men did not. This might be due to gender differences in the uptake of health-related promotion messages [21]. The increased exercise in obese persons is also important since this group can experience significant health benefits from exercise [22].

It is recommended that older adults should have an activity plan for achieving recommended physical activity that integrates preventive and therapeutic recommendations [8]. Different methods to improve exercise among older adults have been tested. Financial incentives tied to aerobic minutes might, for example, be an effective approach for increasing physical activity among sedentary older adults [14].

Sometimes the risk of injury, or rather the fear of injury, is one explanation for why older adults do not exercise. The current literature does not, however, suggest that older adults participating in physical activities are at increased risk of injury [23]. Other reasons for abandoning exercise might be that older adults expect too great an effect too fast and become discouraged when positive effects are not evident. Despite (or due to) these obstacles and because of the overwhelming evidence of the positive effects of exercise, it is important that clinicians offer support to healthy as well as unhealthy individuals to initiate and increase exercise. There is also a need for more studies in this area. There are not many longitudinal studies on exercise in older adults and there is a need for intervention studies as well.

### Study limitations and strengths

This study has some important limitations. One limitation is that our outcome measures were based on self-reported exercise, which may have led to exercise being under- or overestimated [24, 25]. However, if such under- or overestimation exists in our study, it should exist for all three assessments. It is, however, possible that the bias of overestimation caused by self-report is not the same for all assessments, since public awareness of the importance of physical activity may have increased during the study period. Another limitation is that the non-response rate was 20-25%. Non-responders may exercise less than responders to surveys, which may have led to overestimation of the true prevalence of exercise in the population. The reason for not reporting height or weight might be that the person felt that his or her values were outside what they considered a normal range. However, this bias would also be similar for all measurements and should not markedly have affected the longitudinal trends. There might be confounders other than those we adjusted for. For example, we had no data on alcohol consumption and this possible explanatory variable was therefore not adjusted for.

Another limitation of the study is that loss to follow-up may have resulted in selection bias. There were more persons with higher education in the last survey. We have however adjusted for educational level in our analyses. The change in wording in the survey question (in 2004/05 the examples were excluded) must also be considered as an important shortcoming. This may cause an underestimation of physical activity compared to when no examples were given. However, the dichotomization of the question is based on the keyword regular making this shortcoming a little less important. Finally, exercise might have increased because of a survival bias: a greater number of less physically active persons than more physically active persons may have died. The limitations of the present study are, however, counteracted by several strengths. For example, the generalizability of the study is high as it included a representative sample of the whole Swedish population, including all socioeconomic groups. Another strength is the longitudinal design, which is a novel contribution. The same individuals were assessed at intervals of eight years to estimate the annual effects of age group and birth cohort on exercise in the Swedish population during a 16-year period, for each sex separately. Finally, adjustments were made for the possible confounders educational level, self-reported health, smoking, and BMI.

## Conclusions

In this longitudinal study we found an increase in self-reported regular exercise in older adults between 1988/89 and 2004/05. Low prevalence of self-reported regular exercise was associated with poor self-reported health, smoking, obesity, and low educational level. Physical activity promotion in older adults should be of high priority for both primary and secondary prevention of diseases, especially among groups with known risk factors for low levels of exercise.
